# Building Laboratory Capacity to Support the Global Rotavirus Surveillance Network

**Published:** 2013-05-24

**Authors:** Dept of Immunization, Vaccines, and Biologicals, World Health Organization Regional Office for Africa, Regional Office for the Americas, Regional Office for the Eastern Mediterranean, Regional Office for Europe, Regional Office for South-East Asia, Regional Office for the Western Pacific, Fatima Serhan, Div of Viral Diseases, National Center for Immunization and Respiratory Diseases and Global Immunization Div, Center for Global Health, CDC

**Affiliations:** World Health Organization

In 2001, in anticipation of rotavirus vaccine licensure and introduction, the World Health Organization (WHO) and partners established regional laboratory surveillance networks for rotavirus detection and strain type monitoring among hospitalized children aged <5 years (1). In 2006, two WHO-prequalified oral rotavirus vaccines were licensed: a 2-dose, single-strain vaccine (Rotarix, GlaxoSmithKline Biologicals) and a 3-dose, multistrain vaccine (RotaTeq, Merck). Both vaccines provide protection against a range of rotavirus strain types, generally classified as G and P types based on specific viral proteins (2). Based on results of clinical trial data, disease burden data from surveillance networks, and findings from vaccine impact studies, WHO recommends that all countries include rotavirus vaccination in national immunization programs (3). Vaccination is recommended to help reduce the morbidity and mortality associated with rotavirus, a leading cause of diarrhea in children aged <5 years that was responsible for approximately 450,000 deaths in 2008 (4). This report describes the expansion of the regional rotavirus laboratory surveillance networks to a global surveillance network, the implementation of data quality assurance measures to ensure quality laboratory data reporting to support rotavirus surveillance activities, and data reporting through the surveillance network. Timely, quality surveillance data can provide baseline estimates of rotavirus disease burden to inform decisions regarding rotavirus vaccine introduction in national immunization programs and can help monitor the impact of vaccine introduction on disease trends.

## Background

In 2008, the Global Rotavirus Surveillance Network (GRSN) was established to 1) generate local data for decision making regarding rotavirus vaccine introduction and sustained use, 2) assess and monitor disease trends and genotype distribution over time, 3) develop a platform for vaccine effectiveness studies, and 4) highlight the value of surveillance data. The transition of regional laboratory network coordination to WHO for a global laboratory network within the GRSN then began, with GAVI Alliance funding for surveillance in eligible countries. The Global Rotavirus Laboratory Network (GRLN) is a fundamental component of the GRSN designed to conduct high-quality diagnostic testing for rotavirus diarrhea and characterize the most prevalent genotypes among strains isolated in different countries and regions. As of April 2013, the network includes 107 sentinel hospital laboratories, 36 national laboratories, nine regional reference laboratories, and one Global Reference Laboratory.

## Implementation

WHO coordinates the operations of the GRLN and GRSN. WHO surveillance focal persons and laboratory coordinators work closely with ministries of health in participating countries to support surveillance activities, including initial sentinel hospital site selection, specimen and data flow management, laboratory performance monitoring, and regional meeting planning. WHO, partners, and participating countries hold regular meetings at the global and regional levels to discuss surveillance results and progress in network development and to set an agenda of priority activities.

Sentinel hospital sites within participating countries enroll children <5 years of age hospitalized with acute gastroenteritis who meet a standard case definition.* The GRLN, a tiered laboratory structure, supports laboratory testing of stool specimens collected from enrolled children (Figure). Capable sentinel hospital laboratories use antigen-detecting enzyme immunoassay kits to test for presence of rotavirus in stool specimens (5). National laboratories in participating countries provide support to the sentinel hospital laboratories and are responsible for rotavirus testing, specimen storage, and selection and distribution of positive specimens for genotyping (i.e., strain characterization). Rotavirus regional reference laboratories (RRLs), selected for expertise in rotavirus detection and genotyping methods and capacity to provide technical assistance to countries in their region, support the national laboratories. RRLs conduct the bulk of the rotavirus genotyping using reverse transcription–polymerase chain reaction and nucleic acid sequencing methods. However, in some countries, especially larger ones with high sample volume, national laboratories and sometimes sentinel hospital laboratories have genotyping capacity. The Global Reference Laboratory provides technical support to the RRLs, including training in genotyping methods, development and implementation of quality assurance and quality control systems with collaborating partners, provision of standardized laboratory reagents and procedures, and assistance in regional capacity building activities as needed and requested by the RRLs. The Global Reference Laboratory and RRLs also undertake research to improve essential laboratory methods used in the GRSN.

The GRLN has adapted several approaches from the WHO-coordinated global laboratory networks for poliovirus and for measles and rubella to confirm and improve the accuracy of collected data (6,7). These include proficiency testing, standardization of laboratory methods, and laboratory assessments. To help ensure the quality of reported data, a formal external quality assessment program began in 2011 after development of a proficiency testing panel of rotavirus specimens consisting of common genotypes and negative controls. The panel tests the ability of network laboratories to correctly identify positive and negative specimens by antigen enzyme immunoassay and determine the genotypes in positive specimens. Laboratories must achieve a score of at least 80% on each test to pass. In 2011, a proficiency testing pilot survey included nine RRLs from all WHO regions. This was expanded to 10 RRLs, 16 national laboratories, and 17 provincial laboratories during 2012. WHO laboratory coordinators work closely with laboratories with identified weaknesses, based on performance results, to implement corrective actions and improve testing performance.

In 2012, WHO established a rotavirus laboratory technical working group to develop approaches to improve laboratory network capacities and increase standardization of key laboratory methods and procedures. Progress on standardization issues includes recommendations to 1) revise standard genotyping data collection forms to record all detected strains; 2) define approaches to reduce the number of untypeable strains; 3) develop standard procedures for sample handling, storage, and shipping that can be adapted in each region; and 4) implement routine confirmation for a subset of genotypes.

Monitoring of individual laboratory performance occurs through site assessments using standardized assessment tools for the national laboratories and RRLs; a standard tool for sentinel hospital laboratories is in development. Performance indicators for sentinel hospital laboratories and national laboratories include minimum number of rotavirus tests performed, RRL-confirmed testing accuracy, successful completion of yearly proficiency testing, timely sample analysis, and application of standard operating procedures. Additionally, reviewers assess the biosafety procedures and infrastructure of all laboratories. Site visits offer opportunities to assist laboratories with problem solving and often are combined with trainings.

Laboratory data reported through the GRSN include the percentage of hospitalized children positive for rotavirus and strain prevalence in each WHO region and country. The number of reporting countries has grown from 44 in 2008, to 64 in 2011 (8–10). During the same period, the number of participating sentinel hospitals expanded from 132 to 185, and the annual number of enrolled children increased from 41,414 to 48,947. Median global rotavirus detection rates in stool specimens varied from 36% to 41% during 2008–2011; data collection on strain prevalence began in 2009. During 2009–2011, the most frequent genotypes observed were the five considered globally prevalent (G1P[8], G2P[4], G3P[8], G4P[8], and G9P[8]). However, regional differences in genotype prevalence were evident, especially for Africa and South-East Asia where other genotypes constituted a significant proportion of rotavirus genotypes (Table).

## Comment

The GRLN is an integral part of the GRSN that provides timely rotavirus disease burden data, which can help guide decisions regarding rotavirus vaccine introduction into national immunization programs. These data also can provide a baseline for assessing the impact of rotavirus vaccines on severe rotavirus disease resulting in hospitalization and on strain prevalence.

Substantial progress has been made in expanding the reach of the GRLN, developing standardized data collection procedures, and implementing quality assurance procedures to improve data collection. Lessons learned and applied from the other WHO-coordinated laboratory networks have resulted in a system of national, regional, and global laboratories proficient in rotavirus diagnosis and genotyping. Efforts are underway to optimize critical laboratory procedures used at the global and regional reference laboratories to facilitate interlaboratory data comparability and improve genotyping data quality.

Although 2009–2011 data indicate that G1P[8], G2P[4], G3P[8], G4P[8], and G9P[8] strains remain the most prevalent globally, regional and temporal differences in genotypes exist. Strain changes are seen naturally. Careful interpretation is necessary to associate any changes with vaccine use, especially because both vaccines have demonstrated good cross-protection to date. Close monitoring is required and can be accomplished through the GRLN.

WHO, in collaboration with key partners, has begun an in-depth review of the past 5 years of data and experience collected through the GRSN. This review will identify strengths and weaknesses of the GRSN, including the GRLN, and will guide decisions on strategies and actions to ensure the network is responsive to information needs of all immunization stakeholders. The review also will provide recommendations related to the potential use of the network for surveillance needs around vaccines in development and other important gastroenteric pathogens.

## Figures and Tables

**FIGURE:**
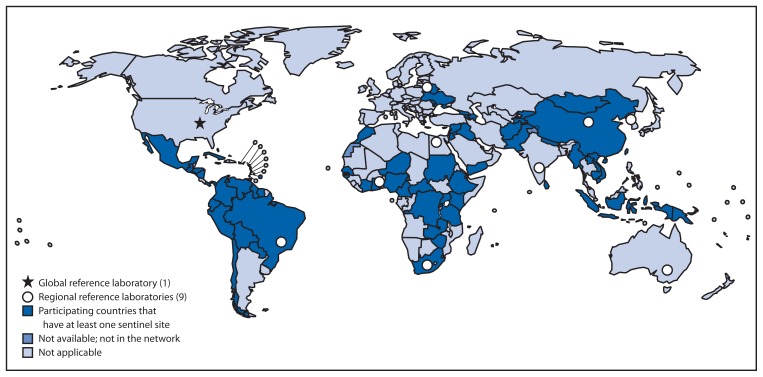
Global Rotavirus Laboratory Network — World Health Organization (WHO), 2013^*^ ^*^ As of March 21, 2013.

**TABLE:** Number and percentage of rotavirus-positive specimens (N = 14,902) from hospitalized patients aged <5 years with rotavirus diarrhea, by strain and World Health Organization (WHO) region — worldwide, 2009–2011

	WHO region												Global	
		
	African		Americas		Eastern Mediterranean		European		South-East Asia		Western Pacific		Total	
							
Strain	No.	(%)	No.	(%)	No.	(%)	No.	(%)	No.	(%)	No.	(%)	No.	(%)
G1P[8]	435	(19)	1,108	(27)	52	(4)	665	(36)	169	(18)	1,965	(47)	**4,394**	**(29)**
G2P[4]	210	(9)	879	(21)	282	(19)	250	(14)	99	(10)	319	(8)	**2,039**	**(14)**
G3P[8]	41	(2)	327	(8)	31	(2)	218	(12)	9	(1)	1,065	(25)	**1,691**	**(11)**
G4P[8]	35	(2)	14	(0)	58	(4)	385	(21)	0	(0)	3	(0)	**495**	**(3)**
G9P[8]	231	(10)	313	(8)	23	(2)	84	(5)	76	(8)	167	(4)	**894**	**(6)**
Others*	759	(33)	1,067	(26)	308	(21)	174	(9)	338	(35)	268	(6)	**2,914**	**(20)**
Mixed†	316	(14)	131	(3)	637	(43)	37	(2)	150	(16)	224	(5)	**1,495**	**(10)**
Untypeable§	246	(11)	322	(8)	94	(6)	35	(2)	112	(12)	171	(4)	**980**	**(7)**
**Total**	**2,273**	**(100)**	**4,161**	**(100)**	**1,485**	**(100)**	**1,848**	**(100)**	**953**	**(100)**	**4,182**	**(100)**	**14,902**	**(100)**

**Source:** World Health Organization. Global rotavirus information and surveillance bulletin. Vols. 2, 4, and 6. Geneva, Switzerland: World Health Organization; 2010, 2011, and 2012. Available at http://www.who.int/nuvi/surveillance/HQBulletin_Rota_2009_final.pdf, http://www.who.int/nuvi/surveillance/Final_RV_bulletin_Jan_Dec_2010_Data.pdf, and http://www.who.int/nuvi/rotavirus/RV_bulletin_Jan_Dec_2011_FINAL.pdf.

* Strains with G and P genotypes other than globally prevalent types G1P[8], G2P[4], G3P[8], G4P[8], and G9P[8]. During 2009 and 2010, the genotypes of these strains were recorded in a data category designated as uncommon and the individual genotypes were not always submitted to WHO. Consequently, only the reported absolute numbers and percentages of these strains are shown. The data table was changed in 2011 so that all genotypes could be recorded and submitted to WHO.

† Strains for which more than one G, P, or G and P genotypes were detected. The individual genotypes of these mixed infections were not always submitted to WHO. Consequently, only the reported absolute numbers and percentages of these strains are shown.

§ Includes strains whose G or P genotype, or G and P genotypes were indeterminate. The individual genotypes of these mixed infections were not always submitted to WHO. Consequently, only the reported absolute numbers and percentages of these strains are shown.

## Footnotes

* Any child aged 0–59 months admitted for treatment of acute (i.e., ≤14 days) watery gastroenteritis/diarrhea to a sentinel hospital conducting surveillance. Excluded are children with bloody diarrhea and children transferred from another hospital.
